# Secretomics to Discover Regulators in Diseases

**DOI:** 10.3390/ijms20163893

**Published:** 2019-08-09

**Authors:** Parkyong Song, Yonghoon Kwon, Jae-Yeol Joo, Do-Geun Kim, Jong Hyuk Yoon

**Affiliations:** 1 Department of Convergence Medicine, Pusan National University School of Medicine, Yangsan 50612, Korea; 2 Department of Life Sciences, Pohang University of Science and Technology, Pohang 37673, Korea; 3 Neurodegenerative Disease Research Group, Korea Brain Research Institute, Daegu 41062, Korea; 4 Dementia Research Group, Korea Brain Research Institute, Daegu 41062, Korea

**Keywords:** cytokine, exosome, LC-MS/MS, proteomics, secretomics, secretome

## Abstract

Secretory proteins play important roles in the cross-talk of individual functional units, including cells. Since secretory proteins are essential for signal transduction, they are closely related with disease development, including metabolic and neural diseases. In metabolic diseases, adipokines, myokines, and hepatokines are secreted from respective organs under specific environmental conditions, and play roles in glucose homeostasis, angiogenesis, and inflammation. In neural diseases, astrocytes and microglia cells secrete cytokines and chemokines that play roles in neurotoxic and neuroprotective responses. Mass spectrometry-based secretome profiling is a powerful strategy to identify and characterize secretory proteins. This strategy involves stepwise processes such as the collection of conditioned medium (CM) containing secretome proteins and concentration of the CM, peptide preparation, mass analysis, database search, and filtering of secretory proteins; each step requires certain conditions to obtain reliable results. Proteomic analysis of extracellular vesicles has become a new research focus for understanding the additional extracellular functions of intracellular proteins. Here, we provide a review of the insights obtained from secretome analyses with regard to disease mechanisms, and highlight the future prospects of this technology. Continued research in this field is expected to provide valuable information on cell-to-cell communication and uncover new pathological mechanisms.

## 1. Introduction

The secretome represents proteins that are secreted from biological units such as organs and cells. Secretory proteins have recently been shown to play important roles in the cross-talk of individual functional units, including facilitating communication between cells [1,2,3]. Since secretory proteins are essential for signal transduction from one locale to another to coordinate biological activities, especially membrane receptor-dependent signal transduction, they are broadly involved in several aspects of biological regulation [4]. In particular, secretory factor-mediated signal transduction largely determines the general cellular fate such as proliferation, growth, migration, and metabolic regulation. In addition to inter- and intra-cellular communication and signal transduction, secretory proteins also play a myriad of functions ranging from roles in the immune system to acting as neurotransmitters in the nervous system [5,6], and have also been suggested to be involved in the building and maintenance of cell membranes [7]. Some secretory proteins have been suggested to act as effectors on pathogens and carry motifs of host cells to avoid detection by the host immune system [8]. The secretory protein structure typically includes an N-terminal and hydrophobic signal peptide, and the proteins are processed via the endoplasmic reticulum (ER) and Golgi apparatus before their eventual secretion into the extracellular space via the classical, non-classical, or exosomal pathways. Furthermore, because these secretory proteins are released into blood plasma, studies of plasma proteins have been of interest [9,10]. Therefore, secretory proteins are widely accepted to play important roles in biological responses and homeostasis of the whole body.

Secretomics is a sub-field of proteomics that represents a powerful strategy for characterizing and quantifying proteins secreted by a given cell under specific conditions. Secretomics is based on two major proteomics workflows: in-solution digestion coupled with LC-MS/MS, and SDS-gel fractionation/in-gel digestion/LC-MS/MS [11,12,13]. Recently, condition-dependent secretome studies have been adopted to discover disease-specific biomarkers or secretory signal regulators. Here, we provide an overview on progress in secretomics techniques, and highlight secretory factors that have been identified with these techniques showing a strong relation to diseases. 

## 2. Overview and Challenges of Secretomics Techniques

The majority of secretome studies in mammalian cells are performed in vitro by first culturing cells of interest in serum-supplemented medium to obtain a sufficient number of cells for evaluation. In this regard, selection of a suitable cell model is an essential factor for determining the composition of the cell secretome that, along with subsequent steps, will result in a reliable proteomic analysis. After culturing in appropriate media, the cells must then be carefully washed with sterile buffered saline to remove any serum contamination, followed by incubation in serum-free medium for certain times depending on the experimental purpose in consideration of maintaining cellular viability. The conditioned medium (CM) containing secreted proteins is generally collected and concentrated for the next step using centrifugal filters. This step can also eliminate contaminant components from the culture medium as well as serum by the addition of buffered saline or ammonium bicarbonate solution [14]. In addition to these conventional techniques, other methods of secretome analysis include the use of resin for cleaning up DNA and concentrating secretory protein mixtures [15] or direct digestion of CM proteins after denaturation using urea and HEPES [6].

After preparation of the secreted proteins, two major proteomics workflows are typically applied to analyze the secretome profiles: in-solution tryptic digestion coupled with LC-MS/MS and SDS-PAGE/in-gel digestion/LC-MS/MS [11,12]. In-solution digestion includes a traditional reduction-alkylation-digestion method, filter-aided sample preparation (FASP), and thermal denaturation-based digestion. Selection of the most appropriate method can be determined according to the biochemical features of the proteomes. Reduction agents include dithiothreitol (DTT), Tris(2-carboxyethyl)phosphine, beta-mercaptorthanol, and alkylation agents, including iodoacetamide, iodoacetic acid, chloroacetamide, and acrylamide [16].

For example, FASP would be selected for a proteome including membrane proteins or cytokine proteins because it uses SDS and urea [17]. Thermal denaturation-based digestion is also suitable for such a proteome because the high temperature required (90 °C) is helpful for denaturing the proteins [18]. In-gel digestion is applicable for high salt-containing protein samples because the salts can be eliminated in the process of electrophoresis [19]. Moreover, in-gel digestion provides high peptide purity, although the extraction efficacy of peptides from the polyacrylamide gel can be low, requiring a relatively larger amount of proteins [20]. In addition, before injection of peptide samples to an LC-MS/MS system, a desalting step using C18 spin tips might be necessary since a high salt concentration will influence the LC results, especially in a trap column, by increasing the pressure [21,22]. The trap column should contain a trap cartridge to remove remaining salts from the samples [23]. There are several conditions that must be considered when applying LC for peptide separation. C18 separation columns are generally selected for reverse-phase chromatography, and both a loading column and separation column are installed [24,25]. There are also diverse specifications of a C18 separation column for nano-LC. C18 columns are available with diverse particle sizes, column diameters, and column lengths; a separation column with a particle size of 2–5 μm and a diameter of 75 μm is generally selected for peptide separation [14,24,26]. Because the column is run at a flow rate of 200–300 nL/min with high back-pressure, the column length and temperature must be optimized to increase the resolution [27]. Use of a column temperature-controlled MS inlet increases the resolution and reproducibility, and allows for the use of even longer columns and/or smaller particle sizes because elevated temperatures lower the viscosity and reduce the overall back-pressure [27,28]. Because secretomes typically contain low-abundant proteins, high-resolution reverse-phase chromatography can help reaching a greater depth of secretome analysis [6,29]. High-resolution chromatography can be achieved using a smaller particle size and longer column length (e.g., 50 cm); this setup allows running longer gradient times for MS, and thus improve resolution and sensitivity [27,30].

After mass analysis, mass raw files are converted to appropriate file formats such as MGF or mzXML, and then compared to a protein database using specially developed search engines, including Mascot, SEQUEST, PEAKS DB, ProteinPilot, pFind, Andromeda, OMSSA, and X!Tandem [31]. Some of the essential search parameters include mass tolerance, miscleavage, digestive enzyme site, fixed (or static) modification, and variable (or dynamic) modifications. Next, quantitative analysis is carried out using label-free or labeling methods. Label-free methods such as those based on an XIC, spectral count, or fragment ion intensity have advantages of being easy to use and providing relatively good accuracy, with reproducible results in biochemical experiments [32,33,34,35]. However, labeling methods such as a tandem mass tag, SILAC, iTRAQ, and TMT are widely used as they provide relatively more accurate quantitative results than label-free methods [36,37,38,39,40].

There are three main methods used to filter secretory proteins from the total identified proteins. First, proteins can be generally identified from Gene Ontology Cellular Components terms of the extracellular region using bioinformatics software such as DAVID bioinformatics resources [41]. Second, secretory proteins are identified based on signal sequence prediction using SignalP and SecretomeP tools [42,43]. The optimal search method and score values should be considered when using these tools. Candidate secreted proteins are indicated by the software with scores values that are calculated according to a prediction model [44]. The secretion of vesicles has also been reported to be involved in the secretome; thus, the ExoCarta exosome database can be selected to screen for putative vesicle-derived proteins [45,46]. 

### 2.1. Cell Culture-based Secretomics

**Figure 1:**
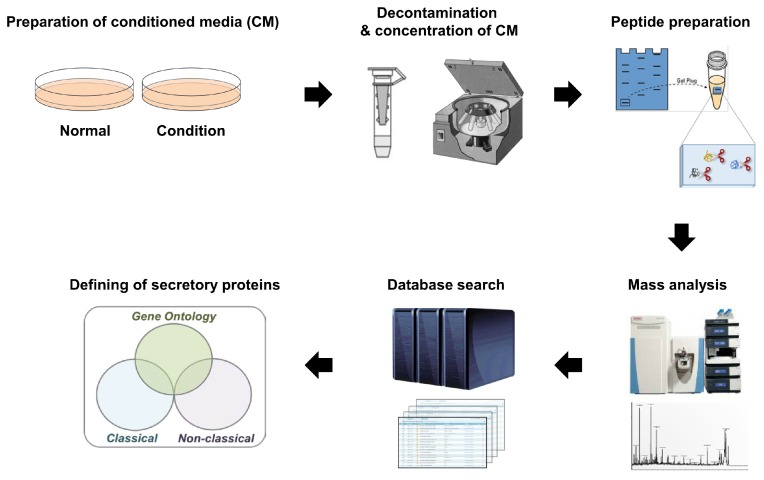
General workflow of cell secretome investigations.

Most secretome studies are performed using a cell culture system. Figure 1 provides a general schematic workflow for a cell secretome study. These studies have provided interesting results contributing to new insights and research directions in diverse fields. Cell culture is a relatively easy method for harvesting secretomes by discriminating expected contaminants such as serum-originated proteins, and for mimicking pathophysiological conditions such as hypoxia, diabetes, and anti-cancer drug treatment [47,48,49]. After culturing cells to appropriate confluence, incubation using serum-free medium is essential to harvest the secretome so as to avoid contamination of serum components [47,48]. However, it has been proposed that a serum-free medium might further contaminate the cell secretory protein profile owing to poor cell viability, thereby reducing the level of capture of the true physiological secretome [47,50]. Depending on the cell type studied, the length of incubation in serum-free medium can vary, which might dramatically influence the secretome profiles [51]. Some cells such as cancer cell lines are very tolerant to serum-free medium, whereas others such as primary cells are more sensitive, which could increase the rate of cell death and the consequent release of intracellular proteins [52]. Therefore, measurement of cellular viability before harvesting the CM is essential for secretome studies with a cell culture system. There are additional options to overcome contaminants from serum that can be labeled or genetically manipulated [53,54]. There is a dual SILAC labeling strategy can exclude unlabeled proteins from serum or cells used for stimulation [53]. Alternatively, another approach involves the selective enrichment of secretory proteins from CM by metabolic marking of newly synthesized glycoproteins via bioorthogonal click chemistry [54]. Only a few studies have attempted to survey the secretome in vivo or ex vivo to date, either by analyzing the secretome of tissue explants without isolating individual cells, or using microdialysis devices [5,55,56].

The new secretory factors identified from cell culture-based secretomics have been reported that are related with diverse disease including metabolic disease, cancer, and neurodegenerative diseases. Irisin has been identified from CM of skeletal muscle cells using LC-MS/MS analysis [57]. Irisin regulates diverse metabolic features, which is discussed in Section 3.1.2. By LC-MS/MS analysis of adipocyte CM, the chemokine ligand 12 (CXCL12) was newly identified as an adipocyte-derived chemotactic factor that has a role in the accumulation of macrophages and production of proinflammatory cytokines in white adipose tissue [58]. Secretomic analysis of skeletal muscle CM under the palmitate induced-insulin resistance revealed that annexin A1 is a new myokine. Following in vitro and in vivo functional study, annexin A1 was identified to play a protective role in the palmitate-induced insulin resistance of L6-myotubes via PKC-theta modulation as well as improved systemic insulin sensitivity in mice fed with a high-fat diet [14]. The hypoxia-induced glioma secretome analysis by LC-MS/MS reported that stanniocalcin 1 (STC1) and stanniocalcin 2 (STC2) have a role in the induction of glioma cell migration in a hypoxia-dependent manner [59]. Neuroregulatory proteins have been also identified and characterized by comparative secretomic analysis of human bone marrow mesenchymal stem cells, adipose tissue derived stem cells and human umbilical cord perivascular cells [60]. Also, therapeutic benefits of CM from mesenchymal stem cells on Parkinson’s disease (PD) has been reported based on secretomic analysis and subsequent functional characterization [61]. In summary, secretomic analysis to discover new regulatory factors are in progress and their application is being expended.

### 2.2. Extracellular Vesicle (EV)-Based Secretomics

Some non-classical secretion mechanisms have been reported with regards to the release of intracellular proteins by cells [62]. Several researchers have proposed mechanisms involving the export of some intracellular proteins to the extracellular compartment, where these proteins usually perform additional extracellular functions that differ from their intracellular roles [63]. Such extracellularly secreted proteins generally exist in Extracellular Vesicles (EVs), a general term to refer to all types of secreted vesicles. EVs are broadly classified into exosomes, microvesicles, and apoptotic bodies according to their cellular origin, size, marker proteins, and functions [64]. Exosomes are nanometer-sized vesicles of endocytic origin that form by inward budding of the limiting membrane of multivesicular endosomes, and were recently shown to facilitate intercellular communication processes between cells in close proximity as well as between more distant cells [64,65]. Diverse cell types such as immune cells, cancer cells, and nerve cells actively secrete EVs along with their own components. EVs stimulate specific responses, including the antigen-presenting response, anti-tumoral immune response, anti-immune response, and myelin formation response according to their molecular contents including cytokines, mRNAs, miRNAs, and lipids [66]. However, the exact role of exosomes is still not clear. Thus, EVs including exosomes are coming into the light of proteomics since they are increasingly targeted in the field of biomedical sciences given elucidation of their various biological and physiological functions.

There are several methods for EVs preparation that are applicable to proteomics, such as ultracentrifugation, immuno-affinity capture, and gel filtration [67,68,69]. Ultracentrifugation-dependent EVs preparation has been the most common method applied because it results in a relatively high yield and is easy to apply [67]. Ultracentrifugation-dependent EVs preparation usually consists of a series of centrifugation cycles with different degrees of centrifugal force and duration to isolate EVs from the secretome based on their density differences [70]. Before the start of ultracentrifugation, a cleaning step is usually carried out to eliminate the CM of large contaminants, including cell debris, using 1000–2000× *g* centrifugation. The sample is then spiked with protease inhibitors to prevent the degradation of vesicle proteins [70,71]. Next, multiple ultracentrifugation steps are carried out with a typical centrifugal force ranging from ~100,000 to 120,000× *g* [70]. During each ultracentrifugation step, pellets are taken for the next step; the final pellets are considered to be the EVs, which are resuspended in an appropriate buffer such as HEPES-buffered saline or PBS [72].

Density-gradient ultracentrifugation has certain advantages in terms of preventing non-vesicular protein contaminants that can be introduced with the conventional ultracentrifugation method [73]. Density-gradient ultracentrifugation involves a high-density solution such as iodixanol: a discontinuous iodixanol gradient (i.e., 40%, 20%, 10%, and 5% *w*/*v*) is generated in ultracentrifugation tubes by sequential layering, and the CM is overlaid on this gradient [74]. Ultracentrifugation is then carried out at 100,000× *g* for 16 h at 4 °C [74]. Each milliliter of the fraction is diluted with a basal buffer such as PBS and then centrifuged again at 100,000× *g* for 2 h at 4 °C, and the resulting pellets are resuspended in PBS [74]. Some exosome preparation kits have also recently been developed, such as ExoQuick and ExoSpin, which are simple and easy to use with reliable results [74,75]. Table 1 is the summary of secretomics techniques.

## 3. Role of Secretory Proteins in Diverse Diseases

### 3.1. Metabolic Diseases

#### 3.1.1. Adipokines

Over 200 adipokines were identified in secretome analyses of adipocytes [76]. Leptin is a representative adipokine that plays a major role as an inflammatory factor in metabolic disorders. The systemic levels of leptin are also positively correlated with body mass index, and are associated with the development of insulin resistance [77]. Leptin has also been shown to affect metabolic signaling pathways of the skeletal muscle: treatment of L6 skeletal muscle cells with recombinant leptin reduced phosphorylation of the insulin receptor substrate-1 and consequently impaired glucose uptake. This observation indicated that leptin promotes insulin resistance [78]. However, other studies showed conflicting results in which leptin increased glucose uptake in other skeletal muscle cells; therefore, further studies are needed to clearly elucidate the function of leptin on the human skeletal muscle [78].

Adiponectin is widely known as a beneficial adipokine because its plasma and protein expression levels are inversely correlated with body weight and abdominal obesity in humans [79]. Adiponectin was also shown to enhance insulin sensitivity in the skeletal muscle, along with elevation of fatty acid oxidation and glucose uptake by activation of AMP-activated protein kinase (AMPK), p38, and peroxisome proliferator-activated receptor (PPAR)-α in skeletal muscle cells [80]. Moreover, adiponectin knockout mice exhibit obese and insulin-resistant phenotypes, whereas systemic administration of adiponectin could improve their insulin sensitivity; these molecular features were also reproducible in human myotubes. Together, these observations indicate that impairment of adiponectin function in the skeletal muscle of obese T2D patients contributes to the development of insulin resistance [81]. 

Under a pathological condition such as severe obesity, the adipose tissue synthesizes and secretes several pro-inflammatory cytokines, which aggravate insulin resistance and the systemic inflammatory status. For example, the tumor necrosis factor-alpha (TNF-α) expression level was shown to be highly increased in adipocytes in a study of obese human subjects [82]. Interestingly, when TNF-α was inhibited by a neutralizing antibody or genetic manipulation, different obesity model mice showed improved insulin sensitivity [83,84]. Similarly, adipose tissue derived interleukin (IL)-1b [85], monocyte chemotactic protein-1 (MCP-1) [86], and chemerin [87] affect local (or systemic) inflammation. Taken together, these findings indicate that the adipose tissue can secrete both beneficial and harmful ligands in a context-dependent manner; thus, a multiple-targeted approach should be considered as the next step in development of an adipokine-based therapeutic strategy for metabolic syndrome.

#### 3.1.2. Myokines

IL-6 is a well-known myokine and popular focus of research into disease mechanisms, particularly in relation to inflammation. IL-6 has been dubbed an “exercise factor” through which skeletal muscles communicate to the peripheral organs. In humans, increased circulating concentrations of IL-6 are known to be affected by both the intensity and duration of skeletal muscle contractions [88]. In vitro experiments with cultured cells showed that IL-6 treatment increases glucose uptake through the AMPK and phosphatidylinosotol 3-kinase (PI3K) pathways [89]. IL-6 treatment was also shown to upregulate glucose uptake regardless of insulin stimulation and glycogen synthesis in healthy myotubes [90]. In humans, IL-6 increases hepatic glucose production and induces whole-body lipolysis [90].

Another important myokine is brain-derived neurotrophic factor (BDNF), which is a member of the neurotrophic factor family. BDNF is also considered to be an exercise factor since its levels are increased as a result of both acute and chronic aerobic exercise [91]. BDNF treatment reduced blood glucose levels in diabetic model mice, and chronic BDNF infusion improved glucose uptake and metabolism in the brown adipose tissue and skeletal muscle of rodents [92]. BDNF was also reported to increase the phosphorylation of AMPK and acetyl-CoA carboxylase (ACC) and to enhance fat oxidation in the skeletal muscle [93]. 

Irisin is a fragment of fibronectin type III domain-containing protein 5 (FNDC5) that was recently reported to act as a myokine, although its origin and function are controversial. Irisin plasma levels are increased by diverse types of exercises; thus, it is also considered to be an exercise hormone. Irisin treatment to L6 myotubes resulted in increased glucose uptake in a dose-dependent manner, which was mediated by activation of both AMPK and ACC. Irisin treatment of primary myocytes also upregulated the expression of PGC-1α4, a specific isoform associated with muscle hypertrophy [94]. Another study showed that irisin treatment upregulated insulin-like growth factor-1 expression and downregulated myostatin expression, suggesting a role in growth of the skeletal muscle [94]. In vivo, administration of irisin to high-fat diet (HFD)-fed mice decreased the fasting blood glucose level, and improved glucose and insulin tolerance; the same effects were detected in obese and HFD-fed mice with FNDC5 overexpression [95]. Irisin released from the skeletal muscle during exercise acts directly on the bone by increasing the cortical bone mineral density, bone perimeter, and polar moment of inertia in mice [96].

Follistatin-like-1 (FSTL-1) is a myokine of the follistatin family that was first identified as a secreted protein from C2C12 mouse myotubes [97]. FSTL-1 is also considered to be an exercise factor since its circulating plasma level is increased in humans following an acute bout of aerobic exercise [98]. Treatment of FSTL-1 to L6 rat myotubes induced glucose uptake via activation of AMPK and calcium calmodulin kinase. Moreover, the FSTL-1-mediated glucose uptake was accompanied by overexpression of glucose transporter 4 (GLUT4) and translocation of GLUT4 to the plasma membrane [99]. Although the exercise factor IL-15 shows similar induction of glucose uptake, this pathway is mediated by the Janus kinase–signal transducer and activation of transcription protein 3 pathway [100]. IL-8 is another exercise-dependent myokine that was also reported as a glucose uptake-inducing factor in C2C12 cells [89]; however, IL-8 is primarily associated with angiogenesis and inflammation [101].

#### 3.1.3. Hepatokines

Fibroblast growth factor 21 (FGF21) belongs to the FGF superfamily and is a key mediator of fatty acid oxidation and lipid metabolism. In contrast to autocrine/paracrine FGF, FGF21 does not have a heparin sulfate-binding domain, and is thus readily released into the circulation and predominantly acts systemically [102]. A transcript-level analysis showed that FGF21 is largely expressed in the pancreas, brown and white adipose tissue, and liver [103]; however, a liver-specific knockout animal study suggested that the liver is the primary source of circulating FGF21 [104]. Moreover, both hepatic and circulating FGF21 levels are elevated in patients with NAFLD and steatohepatitis [105]. Mechanistically, fatty acid-induced PPAR-α activation and ER stress can explain this increase in FGF21 levels in NAFLD [106]. However, contrasting pharmacological effects of FGF21 were observed, resulting in decreased hepatic triglyceride and plasma triglyceride levels, which were associated with weight reduction resulting from increased energy expenditure in ob/ob and HFD-induced obese mice [107]. To explain this apparent contradiction, the authors proposed that increased FGF21 levels represent a protective response against fatty liver disease. A similar phenomenon was observed in the context of alcohol-induced steatosis. The degree of alcoholic fatty liver was enhanced in liver-specific *Fgf21* knockout mice [108]; however, alcohol treatment increased the hepatic FGF21 expression level both in vitro and in vivo [109]. In summary, physiological and pharmacological studies support that FGF21 is induced in hepatocytes by fatty acid or alcohol to alleviate liver damage.

Fetuin-A is an important hepatokine regulating systemic metabolism. Fetuin-A is a phosphorylated glycoprotein that is primarily synthesized by hepatocytes, and was originally characterized as a potent inhibitor of the insulin receptor tyrosine kinase in the liver and skeletal muscle [110]. Fetuin-A knockout mice show increased basal and insulin-stimulated phosphorylation of insulin receptor and improved insulin sensitivity, suggesting that fetuin-A might have a major role in regulating insulin sensitivity [111]. Numerous studies have shown that fetuin-A treatment accelerates systemic inflammatory cytokine levels while reducing adiponectin expression [112]. In line with these findings, fetuin-A levels are increased in patients with NAFLD [113]. Fetuin-A expression seems to be increased by NF-κB and ERK activation, suggesting that inflammatory stimulation itself is an important factor for fetuin-A expression [114]. In turn, fetuin-A acts as an endogenous ligand for Toll-like receptor 4, which is an important pathway for the development of systemic inflammation [115]. By contrast, Li et al. [116] claimed that fetuin-A confers long-lasting protection against lethal systemic inflammation by inhibiting the late pro-inflammatory pathway. Nevertheless, further studies are required to understand the exact role of fetuin-A in the development and progression of inflammation.

Selenoprotein P (SeP) is mainly synthesized in the liver and appears to show upregulated expression in the liver of patients with T2D, NFALD, and cardiovascular disease [117]. Using serial analysis of gene expression and DNA chip analysis, Misu et al. [118] demonstrated that hepatic SeP mRNA expression is correlated with insulin resistance status in humans. Moreover, purified SeP treatment significantly impaired insulin-mediated AKT phosphorylation in hepatocytes and systemic insulin sensitivity, whereas knockdown of SeP in the liver improved glucose intolerance. A similar study was performed with SeP neutralizing antibodies to inhibit SeP function, confirming the improvement of insulin secretion and glucose sensitivity in T2D model mice [119]. Although SeP knockout mice have increased adiponectin levels [120], it is still unclear whether SeP acts as an endocrine factor, such as acting directly on the adipose tissue to modulate adiponectin expression. A tissue-specific SeP receptor knockout study is needed to determine whether there is cross-talk between SeP and the adipokine adiponectin. 

### 3.2. Vascular Diseases

Vascular diseases are conditions that affect vasculature, including arteries and/or veins of the circulatory system. pathological changes of vascular endothelial cells by inflammation play an important role in the development of vascular and heart disease, one group analyzed secretory proteins under TNF-α treatment using LC-MS/MS method [121]. In this study, cytoskeleton and cytoskeleton-binding proteins (tubulin, actin, cofilin, vimentin, elongation factor-1a), membrane-associated proteins involved in intracellular transport (caveolin, annexins), and protein folding (calnexin, calreticulin, isomerase, chaperones) were identified. Also, it has been reported that endothelial progenitor cells (EPCs) derived secretory factors promoted cortical vascular repair after cerebral ischemia [122]. To identify protein candidates, proteome array with EPCs conditioned media has been conducted [123]. As a result, 38 proteins were detected in the media and the author proved that angiogenin is a critical factor for increasing endothelial proliferation. Another study characterized proangiogenic factor thymidine phosphorylase (TP), also known as platelet-derived endothelial cell growth factor (PD-ECGF) in secretome of EPCs throughout comprehensive MALDI-TOF/TOF mass approach [124]. Currently, the relationship between vascular disease progression and these secretory proteins are not elucidated yet. It would be a good approach whether regulation of these proteins can alleviate inflammation mediated vascular diseases.

Vascular smooth muscle cell (VSMC) dysfunction causes major types of vascular disease such as atherosclerosis and hypertension. Recent study identified 349 proteins from VSMC derived microvesicle and exosome [125]. Importantly, the authors of this study compared secreted proteins profile between quiescent and activated VSMCs. Exosomes from activated VSMCs showed increased proteins related to stress response and cell growth regulation (Stat-3, Hsp-70, Peptidyl-prolyl cis–trans isomerase A, Glyceraldehyde-3-phosphate dehydrogenase, Cofilin-2). This observation suggests that exosomes from activated VSMCs can contribute to induction of inflammation and vascular remodeling. Also, aortic smooth muscle cells (ASMC) secretome was analyzed by ESI-MS/MS and the authors identified Hsp-90 as a ROS induced secretory factor [126]. Although these data are promising, pathological effects of these protein candidates are required to be verified in vivo system in the future.

### 3.3. Neural Diseases

#### 3.3.1. Non-Neuronal Cells: Astrocytes and Microglia

Neurons are well-recognized as an important cell population for receiving and transmitting information in the nervous system; however, non-neuronal cell types such as astrocytes and glia are also essential for proper regulation of the nervous system [127]. Importantly, these cells secrete many kinds of proteins that play pivotal physiological and pathological roles in the nervous system, and their abnormal regulation has been associated with the development of various neurodegenerative diseases, including amyotrophic lateral sclerosis and Alzheimer’s disease (AD) [128]. Based on this concept, analysis of the astrocyte and glial secretome would provide essential information about the potential of these cells and their secreted proteins as diagnostic agents or therapeutics for neurological diseases. 

Numerous studies have now provided a comprehensive profile of astrocyte-derived cytokines and secretory proteins [129,130,131]. Han et al. [130] used a murine astrocyte cell line for proteomic and secretomic analysis with a combined two-step digestion and filter-aided sample preparation method, and almost 6000 unique protein groups were identified from the CM of astrocytes. Many of these proteins correlated with well-known astrocyte-mediated cell-to-cell communication pathways such as focal adhesion, extracellular matrix-receptor interaction, and endocytosis. Interestingly, Kyoto Encyclopedia of Genes and Genomes pathway analysis identified that many of the proteins are related to axon guidance, calcium homeostasis, and development of synaptic circuits, which have also been associated with several neurological disorders [132]. To obtain the astrocyte secretome profiles under pathological conditions, Keene et al. [131] analyzed the CM from primary astrocytes treated with inflammatory cytokines to mimic the neuro-inflammatory conditions associated with neurological disorders such as AD and Parkinson’s disease (PD). They found that IL-6, nitric oxide production, cyclooxygenase-2, and nerve growth factor secretion levels increased in the media in response to cytokine treatment. An independent study also demonstrated that human astrocytes produce IL-1β, IL-1ra, TNF-α, IP-10 (CXCL10), and MIP-1α (CCL3) after cytokine treatment [133]. These observations suggest that astrocyte-derived cytokines and chemokines play an important pro-inflammatory (neurotoxic) and neuroprotective role in inflammation-induced neurological disease.

As resident immune cells of the brain, the microglia mediate key functions to support and initiate an immune response to pathogens and damage. Moreover, microglia can regulate neural development and neuronal cell restoration in both healthy and pathological conditions [134]. Microglial activation induces morphological alterations, which consequently changes the surface receptors. Cumulative evidence also points to aging-induced changes in microglial secretory proteins, which is consistent with the concept of a microglia-derived senescence-associated secretory phenotype [135]. Consistently, microglia derived from aged mice exhibit increased basal or lipopolysaccharide-induced expression of TNF-α, IL-1β, and IL-6. Although these cytokines promote clearance of amyloid beta peptide (Aβ) by the microglia in the early stage, therefore protecting against the development of AD, chronic exposure of pro-inflammatory cytokines would suppress the expression of genes involved in Aβ clearance and thus promote the progression of AD [136]. Finally, both beneficial and deleterious functions of microglia-derived cytokines have been reported in the context of prion diseases [137], suggesting that further study is needed to determine the specific factor(s) in addition to exposure time contributing to the microglial activation-induced progression of neurological diseases.

#### 3.3.2. Neural Stem Cells (NSCs)

NSCs are self-renewing, multipotent cells that generate neurons, astrocytes, and glia of the central nervous system. In addition to generation of new graft-derived neurons and glial cells, accumulating evidence indicates that NSC transplants can improve central nervous system diseases, including animal models of multiple sclerosis, AD, and spinal cord injury, by secreting proteins such as growth factors, cytokines, chemokines, metabolites, and bioactive lipids [138].

Yashura et al. [139] used an immortalized human NSC line (HB1.F3) to identify the secreted neurotrophic factors and determine their neuroprotective effects against neurotoxicity. The HB1.F3-derived culture media clearly decreased the degree of 6-OHDA-induced neurotoxicity in vitro. ELISA further demonstrated that stem cell factor and BDNF play important roles in the neuroprotection mechanism. Likewise, NSC transplantation in 6-OHDA-induced experimental PD rats improved behavioral recovery and protected against dopaminergic depletion by increasing the level of glial cell line-derived neurotrophic factor [140]. Demyelination refers to damage to the myelin layer, which is most commonly represented by multiple sclerosis as a demyelinating disease of the CNS. Cuprizone-induced toxicity has been established as an animal model to study demyelination [141]. Transplanted NSCs in this model induced oligodendrocyte progenitor cell proliferation and enhanced remyelination via secretion of platelet-derived growth factor-AA (PDGF-AA), FGF-2, leukemia inhibitory factor, and ciliary neurotrophic factor [142].

Severino et al. [143] adopted a more systematic approach to analyze the NSC secretome using a shotgun LC-MS/MS method. Several of the identified proteins were related to extracellular matrix and cell-adhesion functions, indicating that this type of analysis may have a limitation to characterize soluble factors with established neurotrophic properties. To overcome this problem, they used a commercially available targeted ELISA kit to detect the expression of well-known cytokines and chemokines, and identified a potential role for chemokine (C-C motif) ligand 2 (CCL2, also known as MCP) in neural differentiation. 

### 3.4. Extracellular Vesicles

Extracellular vesicles (EVs) contain proteins and other cellular origin, being used as early pathological condition detection maker in central nervous system (CNS) diseases. Indeed, EVs had been included high level of total Tau, P-T181, P-S396-tau, and Aβ1-42 from fifty-seven Alzheimer’s disease (AD) patients [144]. In particular, several studies have been reported that profiling of RNA expression patterns altered in neurological disorder EVs such as autism spectrum disorder (ASD) [145,146]. In cancer research, Kosaka and colleague reported that EVs mediated interaction of alternation of pathological disorders based on lung cancer and chronic obstructive pulmonary disease (COPD) [147]. Altogether, EVs are strongly involved not only in pathophysiological roles in various human diseases but also provide information for therapeutic potential biomarker.

## 4. Summary and Perspectives

Secretory factors including cytokines such as adipokines, myokines, hepatokines, and EVs are key factors of the secretome, and since their important roles in various diseases are increasingly becoming recognized, the popularity of secretomics analysis is on the rise. Cell-based secretome studies can yield reliable results through diverse and long-term studies, but there are important considerations with regards to secretome preparation. Therefore, optimized and standardized conditions of primary cells are needed for secretome studies, as well as methods for analyzing the secretome at the tissue level, which could provide more direct and reliable results that exclude artifacts. Studies focused on EVs have also recently begun to attract attention. 

One of the major challenges of secretomics is the inherent sensitivity of the mass spectrometer to cover the wide dynamic range for the low abundance of genuine secreted proteins over the high abundant proteins derived from the serum, which is essential for standard maintenance of the sample [148]. High-resolution mass spectrometers such as Q-Exactive HF, in combination with the optimized sample preparation schemes to enrich newly synthesized secreted proteins facilitate identification of low-level secreted protein from the optimized CM for cell growth [47,149,150,151,152]. Advances in the sensitivity of secretome analysis have enabled identifying samples that are highly susceptible to the media condition such as stem cells [153] and primary cells [154], as well as to compare the secreted protein profiles between serum-free and serum-containing CM [47]. Cell-to-cell and organ-to-organ interactions are generally considered to be mediated by ligand-receptor pathways; however, vesicle-mediated interaction is now increasingly recognized. Thus, secretomics research represents a step beyond conventional MS-based proteomics, and is expected to develop into an essential field for understanding inter-cellular and inter-organ signaling, while providing key insight into pathological mechanisms.

## Figures and Tables

**Table 1:** Summary of secretomics techniques.

Category	Method	Advantage	Disadvantage	Application
Digestion	in-solution digestion	- simple process - relatively higher proteome coverage due to no gel-extraction step	- decontamination (desalting) step - low digestion efficacy of hydrophobic proteins	- proteomes containing low abundant proteins such as blood plasma, CSF
	in-gel digestion	- visualization of proteins - high digestion efficacy for hydrophobic proteins by SDS - decontaminated during gel separation-	- time consuming - loss of proteins during destaining - low resolving power to separated proteins - low digestion efficacy & yield - restricted sample throughput	- proteomes containing high abundant proteins with containing SDS or other chemical contaminants
Quantitative analysis	Label-free	- reflect native condition without chemical modification - lower cost	- low accuracy in quantification - no multiplexed analysis - variation on sample preparation	- proteomes containing low abundant proteins - stimulation dependent cellular proteomes
	Label	- multiplexed analysis - no variation in sample preparation - diminished running time - high accuracy in quantification with high protein coverage	- complex sample preparation - increase sample amount - incomplete labeling - high cost - not native condition (artificial) by chemical labeling such as SILAC	- proteomes containing high abundant proteins such as tissue proteomes
EV preparation	Ultracentrifugation	- easy to apply - proteomics & RNA-seq study compatible - high yield	- time consuming - contaminant proteins & nucleic acid may be pelleted	-
	Density gradient	- high purity - RNA-seq study compatible	- time consuming - complex process	-
	Immuno-affinity capture	- easy to apply - no chemical contamination - no need for instruments - proteomics & RNA-seq study compatible	- low yield - small volume only - high reagent cost	-
	Gel filtration	- fast & easy - easy to apply due to single kit - high purity	- low concentrated prep - enrichment step required	-

## Abbreviations

CM	conditioned medium
EVs	Extracellular vesicles
T2D	Type 2 diabetes
AMPK	AMP-activated protein kinase
L6	Immortalized rat skeletal muscle cell line
IL-6	Interleukin 6
ER	endoplasmic reticulum
PI3K	phosphatidylinosotol 3-kinase
BDNF	Brain-derived neurotrophic factor
C2C12	Immortalized mouse myoblast cell line
ACC	Acetyl-CoA carboxylase
HFD	high fat diet
PGC	Peroxisome proliferator-activated receptor gamma coactivator
NAFLD	nonalcoholic fatty liver disease
FGF21	Fibroblast growth factor 21
GLUT4	glucose transporter 4
FASP	filter-aided sample preparation
TNF-α	tumor necrosis factor-alpha
MCP	monocyte chemotactic protein
FNDC5	Fibronectin type III domain-containing protein 5
IP-10	Interferon gamma-induced protein 10
FSTL-1	Follistatin-like-1
GLUT4	Glucose transporter 4
MIP	Macrophage inflammatory protein
SeP	Selenoprotein P
NSC	Neural stem cell
LC-MS/MS	Liquid Chromatography with tandem mass spectrometry
SILAC	stable isotope labeling by amino acids in cell culture
iTRAQ	isobaric tags for relative and absolute quantification
TMT	tandem mass tag
IL-1β	interleukin 1 beta
VSMC	vascular smooth muscle cell
EPC	endothelial progenitor cells
